# A practical nomogram and risk stratification system predicting the cancer-specific survival for patients aged >50 with advanced melanoma

**DOI:** 10.3389/fonc.2023.1166877

**Published:** 2023-07-13

**Authors:** Qiufen Xi, Xiaoou Lu, Jiali Zhang, Da Wang, Yu Sun, Hongquan Chen

**Affiliations:** Department of Dermatology, The Affiliated Hospital of Qingdao University, Qingdao, China

**Keywords:** advanced melanoma, SEER, nomogram, classification system, AJCC staging

## Abstract

**Objective:**

To investigate risk factors for advanced melanoma over 50 years of age and to develop and validate a new line chart and classification system.

**Methods:**

The SEER database was screened for patients diagnosed with advanced melanoma from 2010 to 2019 and Cox regression analysis was applied to select variables affecting patient prognosis. The area under curve (AUC), relative operating characteristic curve (ROC), Consistency index (C-index), decision curve analysis (DCA), and survival calibration curves were used to verify the accuracy and utility of the model and to compare it with traditional AJCC tumor staging. The Kaplan-Meier curve was applied to compare the risk stratification between the model and traditional AJCC tumor staging.

**Results:**

A total of 5166 patients were included in the study. Surgery, age, gender, tumor thickness, ulceration, the number of primary melanomas, M stage and N stage were the independent prognostic factors of CSS in patients with advanced melanoma (P<0.05). The predictive nomogram model was constructed and validated. The C-index values obtained from the training and validation cohorts were 0.732 (95%CI: 0.717-0.742) and 0.741 (95%CI: 0.732-0.751). Based on the observation and analysis results of the ROC curve, survival calibration curve, NRI, and IDI, the constructed prognosis model can accurately predict the prognosis of advanced melanoma and performs well in internal verification. The DCA curve verifies the practicability of the model. Compared with the traditional AJCC staging, the risk stratification in the model has a better identification ability for patients in different risk groups.

**Conclusion:**

The nomogram of advanced melanoma and the new classification system were successfully established and verified, which can provide a practical tool for individualized clinical management of patients.

## 1 Background

The malignant transformation of melanocytes gives rise to the highly aggressive malignant tumor known as melanoma (1). Lymphocyte and hematogenous metastasis can occur in the early stage, with poor prognosis. Its incidence rate increases year by year all over the world, especially in western countries (2). The mean age of onset was 45 years old and increased with age after 50. Although melanoma accounts for only 5% of skin cancers, it has the highest mortality among skin cancers and is the most serious type (3–5). Skin melanoma is the primary subtype in the west, and most patients are in the early stage of diagnosis. Patients with early melanoma obtain a good prognosis by surgical resection of the primary lesion (6). However, for advanced melanoma, especially stage IV melanoma, studies have found that the average five-year survival rate is less than 10%, and the median progression-free survival period is only 1.7 months (7, 8). As a result, the focus and direction of research continue to be on the prognosis and therapy of advanced melanoma.

The prognosis of malignant melanoma is closely related to tumor staging. At present, the 8th edition of the American Joint Committee on Cancer (AJCC) staging system (9), jointly developed by the American Cancer Society and the union for international cancer control (UICC), is widely adopted. The system is based on assessing the primary tumor, regional lymph nodes and lymphatic drainage, and the presence or absence of distant metastasis without considering other clinically significant prognostic markers, including age, gender, ethnicity, and anatomic sites (10–15). Therefore, a more personalized and comprehensive prediction model is needed to predict the prognosis of patients with advanced melanoma.

Prognostic models of nomogram have been established for a variety of tumors (15–18). As a clinical prediction tool, the individual probability of clinical events can be generated by integrating different prognoses and determinant variables, which helps to make a personal prediction on the survival rate of patients (18, 19) and promotes personalized medical treatment (20, 21). Through a retrospective review of the SEER database, this study developed a prognostic nomogram and a new classification system for advanced melanoma in the current study, all while internally verifying their accuracy.

## 2 Information and methods

### 2.1 The choice of patients

**Figure 1:**
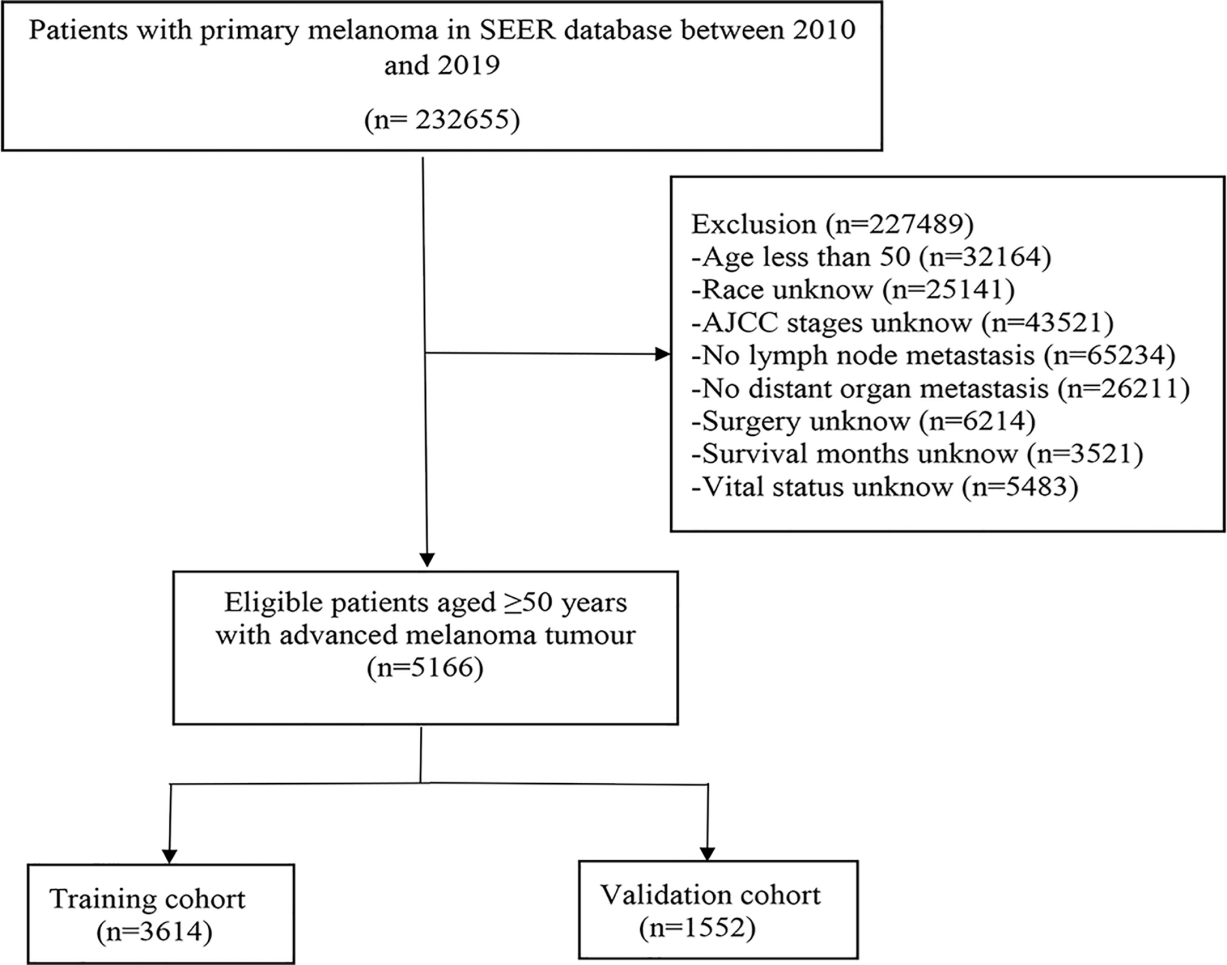
The flowchart of the patients aged >50 with advanced melanoma.

Using SEER*STata, 5,166 eligible patients with advanced melanoma were selected from the SEER database. Inclusion criteria were as follows: (1) The diagnosis was made from 2010 to 2019; (2) The tissue type code of the International Classification of Tumor Diseases (ICD-O-3) III is 8720-8799, and the anatomical site code of the primary tumor is C 44.0-C 44.9; (3) The first primary tumor. Exclusion criteria: (1) Clinical information is incomplete, such as race, lymph node status, distant organ metastasis status, tumor stage (based on AJCC stage), and the operation type is unknown; (2) The sources of patient reports are limited to autopsy and death certificate; (3) The cause of death is unknown; (4) Follow-up survival time unknown; (5) age < 50 years old. See the flow chart for the specific screening process (**Figure 1**).

### 2.2 Building the nomograms

The 5166 samples were randomly allocated into training and validation cohorts in a 7: 3 ratio. The training cohort is used to filter variables and build the model. The validation cohort is used to verify the results obtained using the training cohort. Based on clinical experience and literature review, the following 10 variables were considered possible prognostic influencing factors: age, gender, race, tumor location, tumor thickness, ulceration, N stage, M stage, the number of primary melanomas, and surgery. Tumor staging is based on the Version 8 AJCC Staging System. Prognostic nomogram was constructed based on the variables identified from the univariate and multivariate Cox regression analyses (P<0.05).

### 2.3 Validation the nomograms

The results of a multivariate Cox regression analysis were applied to establish the nomogram to predict the probability of tumor specific survival (CSS) at 3, 6 and 9 years for melanoma patients. The C-index, time-dependent ROC and calibration plots were employed to evaluate the performance of the model. C-index and AUC values with 0.50-0.70 being less accurate, Moderate accuracy between 0.71-0.90, and Above 0.90 being high accuracy. Values greater than 0.7 for C-index and AUC are generally considered good nomograms. The calibration curves were applied to evaluate the difference between the predicted and actual values of the model. The Net Reclassification Index (NRI), Integrated Discriminant Improvement (IDI) and Decision Curve Analysis (DCA) were utilized to demonstrate the strengths and weaknesses of the different performances of the nomogram compared to the AJCC staging system. NRI, IDI are applied to demonstrate the degree of predictive improvement of the nomogram relative to AJCC staging, and to measure the ability to apply them. DCA curves indicated the magnitude of the net clinical benefit of the nomogram compared to each model.

### 2.4 Establishment of a new risk classification system and comparison with AJCC staging

We calculated the total points for all patients based on the nomograms’ scores. Based on the X-tile software, the optimal cut-off point for patient risk score was selected to divide all patients into low-risk, middle-risk, and high-risk groups. The Kaplan-Meier survival curve was used to compare the difference between the newly established risk classification system and AJCC staging in predicting the prognosis and survival of patients.

### 2.5 Data analysis

The endpoint in this study was patient-specific survival for melanoma (CSS), which is the time from diagnosis to death due to melanoma in tumor patients. The data were extracted using SEER*Stat software (8.3.9.2 version), and the best cut-off value for the total points was selected using X-Tile (version 3.6.1). All data analyses were performed using the R software version 4.1.2 (http://www.r-project.org/). The nomograms were developed and validated with R packages “regplot,” “mstate,” “survival,” “cmprsk,” “Hmisc,” “timeROC,” “foreign,” “nricens,” “rmda,” and “DCA.” Chi-square test was used to assess the differences of distribution of the two cohorts. Bidirectional probability values P < 0.05 were considered statistically significant.

## 3 Results

### 3.1 Characteristics of patients and diseases

A total of 5,166 patients with advanced melanoma were enrolled and randomized 7:3 into a training group (3,614) and a validation group (1,552). What is the median follow-up time for the entire population 26 months [Quartile range (IQR): (11-66) months, Training cohort 27 months (IQR): 11-66 months, Validation cohort 26 months (IQR): (12-66.75) months. This study summarizes the demographic and clinical features of advanced melanoma patients > 50 years of age (**Table 1**). The demographic and clinical characteristics of the training and validation cohorts were comparable. (P>0.05).

**Table 1:** Demographics, clinical characteristics of patients aged >50 with advanced melanoma.

Variable	Whole population		Training cohort		Validation cohort		P value
	n	%	n	%	n	%	
	5166		3614		1552		
Age	0						
50-60	1696	32.83%	1183	32.73%	513	33.05%	0.372
60-75	2540	49.17%	1795	49.67%	745	48.00%	
>75	930	18.00%	636	17.60%	294	18.94%	
Race							
Black	68	1.32%	55	1.52%	13	0.84%	0.124
White	4990	96.59%	3477	96.21%	1513	97.49%	
Other	108	2.09%	82	2.27%	26	1.68%	
Sex							
F	1743	33.74%	1226	33.92%	517	33.31%	0.591
M	3423	66.26%	2388	66.08%	1035	66.68%	
Thickness							
0-1	1102	21.33%	778	21.53%	324	20.88%	0.633
1-2	1214	23.50%	860	23.80%	354	22.81%	
2-4	1452	28.11%	1000	27.67%	452	29.12%	
>4	1398	27.06%	976	27.01%	422	27.19%	
Ulceration							
Yes	2631	50.93%	1828	50.58%	803	51.74%	0.482
No	2535	49.07%	1786	49.42%	749	48.26%	
Number							
1	4435	85.85%	3100	85.78%	1335	86.02%	0.752
>1	731	14.15%	514	14.22%	217	13.98%	
Stage_M							
No	4756	92.06%	3328	92.09%	1428	92.01%	0.891
Yes	410	7.94%	286	7.91%	124	7.99%	
Stage_N							
N1	3033	58.71%	2136	59.10%	897	57.80%	0.532
N2	1388	26.87%	960	26.56%	428	27.58%	
N3	745	14.42%	518	14.33%	227	14.63%	
Location							
Head and face	832	16.11%	578	15.99%	254	16.37%	0.704
Trunk	1804	34.92%	1279	35.39%	525	33.83%	
Upper limb and shoulder	1124	21.76%	768	21.25%	356	22.94%	
Lower limb and hip	1276	24.70%	896	24.79%	380	24.48%	
Others	130	2.52%	93	2.57%	37	2.38%	
AJCC Stages^a^							
III	4362	84.44%	3047	84.31%	1315	84.73%	0.622
IV	804	15.56%	567	15.69%	237	15.27%	
Chemotherapy							
Yes	479	9.27%	344	9.52%	135	8.70%	0.411
No	4687	90.73%	3270	90.48%	1417	91.30%	
Surgery							
No	1395	27.00%	988	27.34%	407	26.22%	0.383
Yes	3771	73.00%	2626	72.66%	1145	73.78%	

a AJCC Stages: The eighth edition American Joint Committee on Cancer (AJCC) TNM staging system.

### 3.2 Univariate and multivariate analysis

In the univariate regression analysis, age, race, gender, tumor thickness, ulceration, the number of primary melanomas, M stage, N stage, anatomic site, and whether to have surgery. were the prognostic factors for patients with advanced melanoma (P<0.05). After multivariate analysis of these factors, it was concluded that surgery, age, gender, tumor thickness, ulceration, the number of primary melanomas, M stage, and N stage were the independent prognostic factors for CSS in patients with advanced melanoma (P < 0.05). The above factors were included, and the nomogram was constructed (**Table 2**).

**Table 2:** The results of univariate and multivariate Cox regression analyses.

Variable	Univariate		P value	Multivariate		P value
	HR	95%CI		HR	95%CI	
Age						
50-60	Reference			Reference		
60-75	1.37	1.20-1.57	<0.001	1.40	1.22-1.62	<0.001
>75	2.56	2.19-3.00	<0.001	2.38	2.02-2.80	<0.001
Race						
Black	Reference			Reference		
White	0.52	0.36-0.75	0.025	0.89	0.67-1.25	0.453
Other	0.78	0.47-1.28	0.333	1.01	0.61-1.67	0.671
Sex						
F	Reference			Reference		
M	1.23	1.09-1.40	0.003	1.21	1.06-1.38	0.003
Thickness						
0-1	Reference			Reference		
1-2	1.21	1.05-1.47	0.034	1.12	0.85-1.37	0.541
2-4	1.68	1.38-2.04	0.029	1.17	1.05-1.43	0.049
>4	2.78	2.40-3.20	<0.001	1.28	1.12-1.55	<0.001
Ulceration						
Yes	Reference			Reference		
No	0.44	0.39-0.49	<0.001	0.56	0.49-0.63	<0.001
Number						
1	Reference			Reference		
>1	0.57	0.48-0.68	<0.001	0.61	0.51-0.73	<0.001
Stage_M ^a^						
No						
Yes	4.22	3.60-4.95	<0.001	2.95	2.50-3.48	<0.001
Stage_N						
N1	Reference			Reference		
N2	1.38	1.21-1.58	<0.001	1.27	1.11-1.45	<0.001
N3	2.77	2.40-3.20	<0.001	2.00	1.72-2.32	<0.001
Location						
Head and face	Reference			Reference		
Trunk	0.89	0.75-1.05	0.172	1.09	0.92-1.29	0.341
Upper limb and shoulder	0.81	0.67-0.98	0.048	0.98	0.81-1.18	0.857
Lower limb and hip	0.89	0.74-1.06	0.202	0.89	0.74-1.08	0.263
Others	1.10	0.76-1.58	0.584	1.22	0.85-1.76	0.272
Surgery						
No	Reference			Reference		
Yes	0.72	0.63-0.81	<0.001	0.80	0.71-0.91	<0.001

a AJCC Stages: The eighth edition American Joint Committee on Cancer (AJCC) TNM staging system.

### 3.3 Construction and verification of nomograms

**Figure 2:**
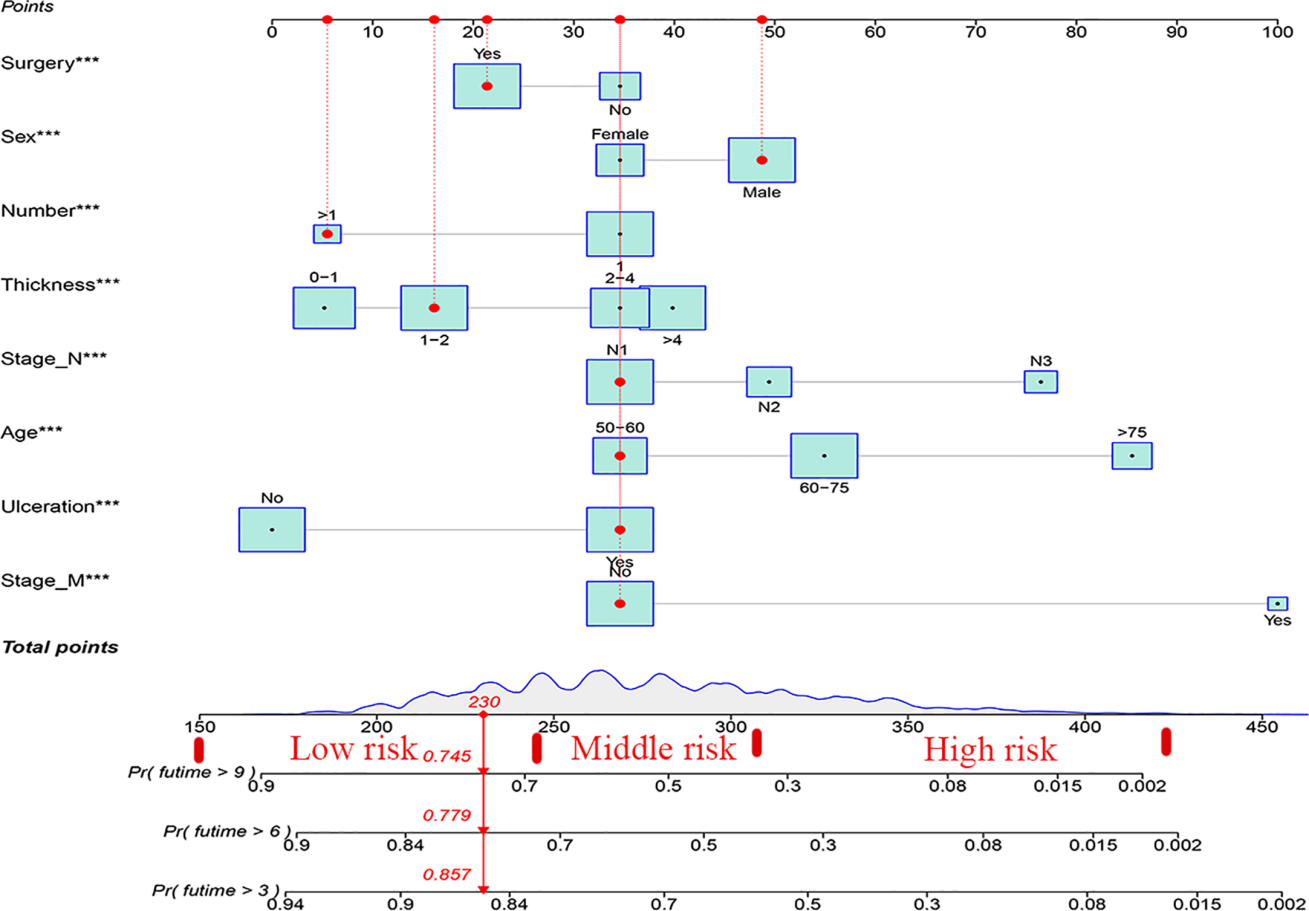
The nomogram for patients aged >50 with advanced melanoma. *** P < 0.001.

**Figure 3:**
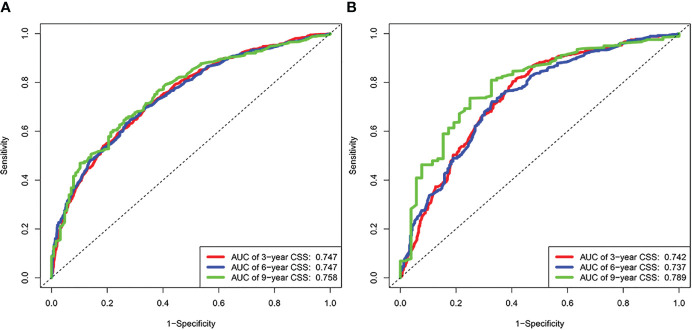
Results of time dependent ROC curve analysis based on the nomogram. **(A)** Based on the training cohorts; **(B)** Based on the validation cohorts.

**Figure 4:**
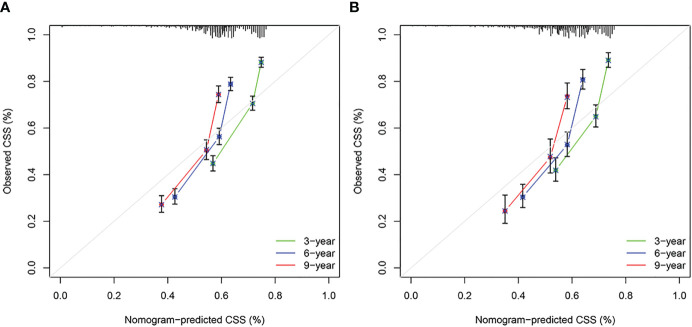
Calibration curves. **(A)** Based on the training cohorts; **(B)** Based on the validation cohorts.

**Figure 5:**
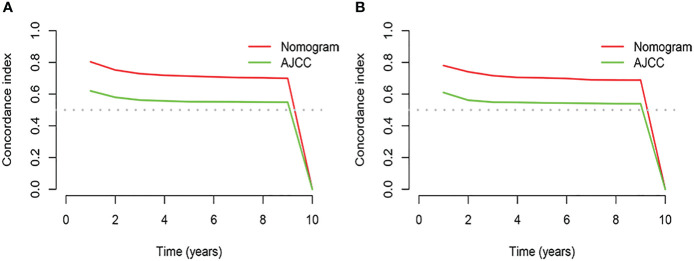
C-index results. **(A)** Based on the training cohorts; **(B)** Based on the validation cohorts.

Based on the results of univariate and multivariate analyses, surgery, age, gender, tumor thickness, ulceration, number of primary melanoma, M-stage, and N-stage were included in the construction of the nomograms for the CSS probabilities of patients with advanced melanoma (**Figure 2**). The C-index, calibration curve, ROC curve, and DCA curve are shown in **Figures 3**–**5**. The C-index for the training and validation cohorts were 0.732 (95%CI: 0.717-0.742) and 0.741(95%CI: 0.732-0.751), respectively. The results of ROC curve analysis showed that the AUCs of the training cohort were 0.747,0.747, and 0.758 at 3, 6, and 9 years, respectively. The AUCs of the validation cohort were 0.742,0.737, and 0.789 at 3, 6, and 9 years, respectively, indicating that the model had good prediction performance. Additionally, calibration curves showed high agreement between predicted and observed CSS rates at 3, 6, and 9 years in both the training and validation cohorts.

### 3.4 Comparison of clinical values of nomograms and AJCC staging

**Figure 6:**
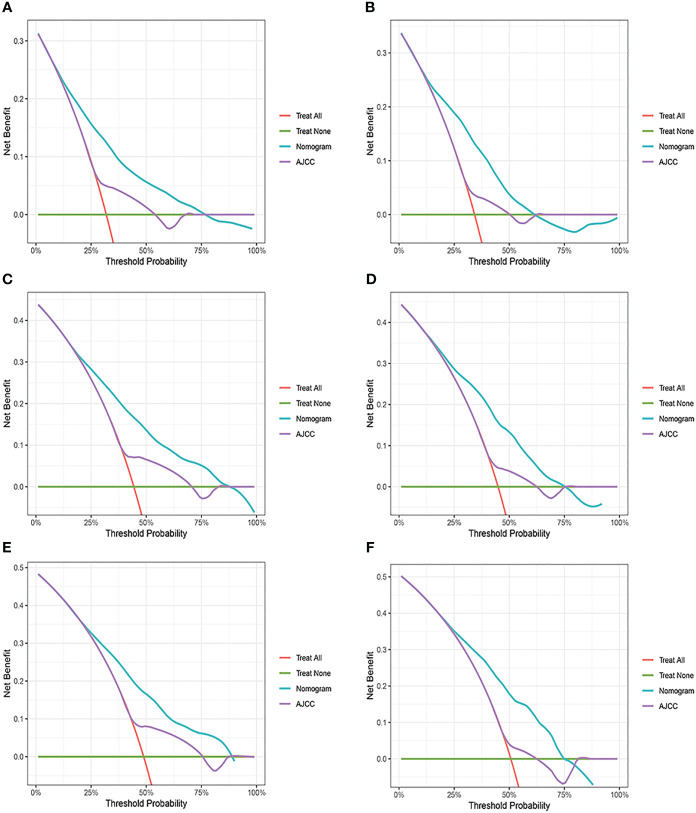
Decision curve analysis of the nomogram and AJCC tumor staging. **(A)** 3-year survival benefit in the training cohort. **(B)** 3-year survival benefit in the validation cohort. **(C)** 6-year survival benefit in the training cohort. **(D)** 6-year survival benefit in the validation cohort. **(E)** 9-year survival benefit in the training cohort. **(F)** 9-year survival benefit in the validation cohort.

Changes in C-index, NRI, IDI, and DCA were used to compare the difference in nomograms with the ability to predict based on AJCC criteria. The nomogram-related C-index was higher in the training and validation cohorts than in the AJCC staging-related C-index. As can be seen from the DCA curve, the nomograms showed a much greater net gain compared to the AJCC standard tumor staging and the two extremes (**Figure 6**). The NRI of the 3-,6-, and 9-year CSS for the training cohort was 0.69 (95%CI=0.65-0.82), 0.70 (95%CI=0.62-0.77), and 0.66 (95%CI=0.57-0.74, and the IDI was 0.12 (95%CI=0.09-0.14), 0.14 (95%CI=0.12-0.17), and 0.16 (95%CI=0.12-0.19), respectively. These results were presented in the validation cohort (**Table 3**).

**Table 3:** NRI and IDI to evaluate the predictive power of the model.

Index	Training cohort		*P* value	Validation cohort		*P* value
	Value	95%CI		Value	95%CI	
NRI						
**3-year CSS**	0.69	0.65-0.82		0.71	0.60-0.84	
**6-year CSS**	0.70	0.62-0.77		0.72	0.60-0.82	
**9-year CSS**	0.66	0.57-0.74		0.68	0.57-0.84	
IDI						
**3-year CSS**	0.12	0.09-0.14	<0.001	0.11	0.07-0.14	<0.001
**6-year CSS**	0.14	0.12-0.17	<0.001	0.13	0.10-0.16	<0.001
**9-year CSS**	0.16	0.12-0.19	<0.001	0.15	0.10-0.21	<0.001

### 3.5 Risk stratification of nomograms

**Figure 7:**
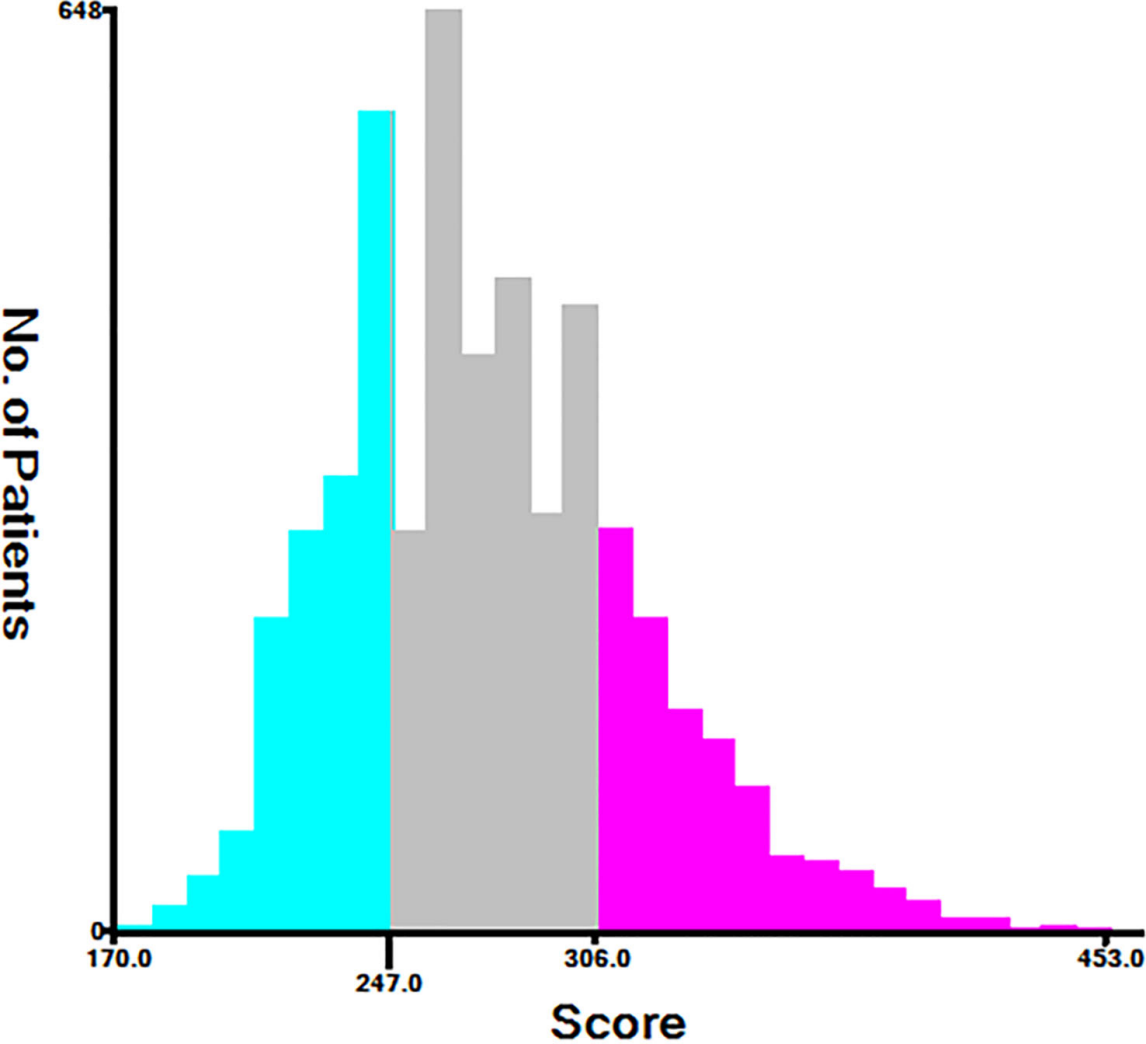
Cut-off point selected using X-tile.

**Figure 8:**
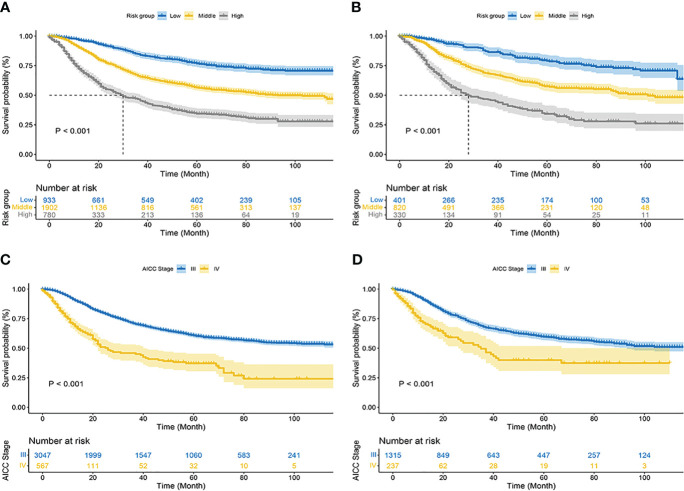
Kaplan–Meier curves of cancer-specific survival for new risk classification and the AJCC tumor staging. **(A)** The new risk classification in the training cohort; **(B)** The new risk classification in the validation cohort; **(C)** The AJCC tumor staging in the training cohort; **(D)** The AJCC tumor staging in the validation cohort.

Using nomograms, total points are calculated and used to stratify risk. Three risk groups were formed for patients with advanced melanoma: low risk (total points < 247.0), middle risk (247.0≤ total points < 306.0) and high risk (total points ≥306.0) (**Figure 7**). The survival curve illustrated the ability of the new risk classification system to clearly distinguish between low and high-risk groups compared to AJCC staging, which will provide a practical tool for the clinical management of patients with advanced melanoma. (**Figure 8**).

## 4 Discussion

Melanoma is a highly malignant neoplasm with a much lower incidence of skin cancer than other skin cancers, such as basal cell carcinoma (70%) and squamous cell carcinoma (25%). Still, melanoma accounts for the majority of skin cancer deaths (22). Like most malignant tumors, the stage of melanoma determines its prognosis and directly affects the choice of treatment plan for patients. Although breakthrough has been made in chemotherapy (23), immunotherapy (24, 25), targeted therapy (26), and other therapies in recent years, surgery and pathological examination are still the first choice for diagnosis and treatment (27). Now that the survival rate of elderly patients with advanced melanoma is still low, which is highly harmful to human beings, accurate prognosis prediction is crucial for better management of elderly patients with advanced melanoma. The AJCC staging system is currently widely used for melanoma staging. The new version of the revision focuses more on the evidence-based revision of stage I to stage III melanoma without establishing a stage IV database or analysis for patients with stage IV melanoma (9). Second, biological factors such as age, gender, and ethnicity, independent risk factors for melanoma, were not included in the AJCC system due to their limitations (28, 29). Only using TNM staging to guide the treatment and prognosis of patients cannot rule out individual differences of patients, which will cause difficulties for personalized medicine.

The predictive nomogram model has been shown to be superior to traditional staging systems in various cancers (30–33). We constructed a nomogram to predict the prognosis of patients with advanced melanoma. Eight variables were selected and incorporated into the nomogram. The 3-, 6- and 9-year survival rates of the patients can be calculated by the scores of the corresponding factors in the nomogram to guide clinical practice. Our nomogram indicates that a poorer prognosis in patients is associated with male gender, absence of surgery, advanced age, increased tumor thickness, concurrent tumor ulceration, and later N and M stages. The above conclusions are consistent with the results in the existing studies (34–36). Currently, the effect of the number of primary melanomas on the prognosis of melanoma is still controversial (37–43).. Based on samples from SEER database 5166, this study found that patients with a single primary melanoma had a worse prognosis according to both univariate and multivariate Cox regression analyses, which is consistent with some existing studies. In addition, the nomogram was constructed utilizing a substantial sample size, resulting in a relatively comprehensive prognostic evaluation for melanoma that is not limited to a single factor. The predictive and calibration abilities of the nomogram have also been confirmed through multi-angle validation.

In this study, several limitations were identified. First, it is important to note that the SEER database has certain limitations of its own. Specifically, the database lacks comprehensive data on newer treatment modalities, such as immunotherapy and targeted therapy. Furthermore, the database does not categorize primary types of melanomas with great specificity, which may limit the current clinical relevance of diagnosis and treatment plan. Secondly, retrospective data collection may inherently introduce certain biases, and the exclusion of patients with missing data could result in a selection bias. Thirdly, we randomly divided the enrolled patients into a training cohort and a validation cohort according to the ratio of 7: 3 and developed a nomogram for internal validation. While the C-index and AUC values obtained were relatively high, and the risk stratification of the nomogram was superior to the AJCC stage prediction model, it is important to note that the data used for modeling and calibration are from the same database, which limits the model’s generalizability. Finally, while the nomograms developed in this study have not been tested in clinical trials, their accuracy and utility remain controversial. Therefore, further research is required to confirm the findings of this study, particularly through randomized clinical trials that can serve as a gold standard for evaluating the performance of the nomograms.

## 5 Conclusion

The nomogram and risk classification system for patients over 50 years of age with advanced cutaneous melanoma based on data from the SEER database, which can help develop personalized treatment options for these patients. However, its clinical utility needs to be evaluated, which is also where the nomogram needs further improvement.

## Data availability statement

The original contributions presented in the study are included in the article/**Supplementary Material**. Further inquiries can be directed to the corresponding author.

## Author contributions

Data analysis were performed by QX. The manuscript was written by QX and revised by HC. All authors contributed to the article and approved the submitted version.
